# Circular RNA circGSE1 promotes angiogenesis in ageing mice by targeting the miR-323-5p/NRP1 axis

**DOI:** 10.18632/aging.203988

**Published:** 2022-04-01

**Authors:** Jiacong Qiu, Rencong Chen, Lei Zhao, Chong Lian, Zhen Liu, Xiaonan Zhu, Jin Cui, Siwen Wang, Mingshan Wang, Yingxiong Huang, Shenming Wang, Jinsong Wang

**Affiliations:** 1 Division of Vascular Surgery, The First Affiliated Hospital, Sun Yat-Sen University, Guangzhou 510080, Guangdong, China; 2 National-Local Joint Engineering Laboratory of Vascular Disease Treatment, Guangzhou 510080, Guangdong, China; 3 Guangdong Engineering and Technology Center for Diagnosis and Treatment of Vascular Diseases, Guangzhou 510080, Guangdong, China; 4 Department of Endovascular Surgery, The First Affiliated Hospital of Zhengzhou University, Zhengzhou 450000, Henan, China; 5 Department of Pharmacology Laboratory, Zhongshan School of Medicine, Sun Yat-Sen University, Guangzhou 510080, Guangdong, China; 6 Department of Emergency, The First Affiliated Hospital, Sun Yat-Sen University, Guangzhou 510080, Guangdong, China

**Keywords:** ageing, angiogenesis, endothelial cell, circGSE1, NRP1

## Abstract

Age is an important factor in many cardiovascular diseases, in which endothelial cells (ECs) play an important role. Circular RNAs (circRNAs) have been reported in many cardiovascular diseases, but their role in ageing EC-related angiogenesis is unclear. We aimed to identify a functional circRNA that regulates angiogenesis during ageing and explore its specific mechanism. In this study, we searched for differentially expressed circRNAs in old endothelial cells (OECs) and young endothelial cells (YECs) by circRNA sequencing and found that circGSE1 was significantly downregulated in OECs. Our study showed that circGSE1 could promote the proliferation, migration and tube formation of OECs *in vitro*. In a mouse model of femoral artery ligation and ischemia, circGSE1 promoted blood flow recovery and angiogenesis in the ischemic limbs of ageing mice. Mechanistically, we found that overexpressing circGSE1 reduced miR-323-5p expression, increased neuropilin-1 (NRP1) expression, and promoted proliferation, migration, and tube formation in OECs, while knocking down circGSE1 increased miR-323-5p expression, reduced NRP1 expression, and inhibited proliferation, migration, and tube formation in YECs. During EC ageing, circGSE1 may act through the miR-323-5p/NRP1 axis and promote endothelial angiogenesis in mice. Finally, the circGSE1/miR-323-5p/NRP1 axis could serve as a potential and promising therapeutic target for angiogenesis during ageing.

## INTRODUCTION

Angiogenesis refers to the formation of new blood vessels from existing capillaries or postcapillary veins and is a complex physiological response in which the activation, proliferation and migration of vascular ECs play a pivotal role [1, 2]. Ageing significantly affects the physiological characteristics of arteries and is an important factor in peripheral and coronary artery disease [3, 4]. Moreover, ageing has a considerable effect on angiogenesis [5, 6].

Increasing evidence suggests that circRNAs, a special class of RNA molecules with a closed-loop structure, play an essential role in the regulation of angiogenesis and in the occurrence and development of many cardiovascular diseases, including atherosclerosis, myocardial infarction, diabetic retinopathy and cancers [7–12], and age is an important factor in the development of these diseases. CircRNAs have been shown to regulate cell functions by acting as microRNA sponges, RNA-binding proteins, nuclear transcription regulators, and templates that are directly translated into proteins [10, 13–15]. While a few studies have been reported on the roles of circRNAs in ageing, their roles in EC senescence and the underlying mechanism have not been well elucidated [16].

NRP1 is a transmembrane glycoprotein that is expressed on blood vessels, neurons, immune cells, and many other types of mammalian cells and binds a range of structurally and functionally diverse extracellular ligands to regulate organ development and function [17, 18]. NRP1 plays a pivotal role in EC angiogenesis [19], and the VEGFR2/NRP1 complex increases the binding affinity of VEGF165 by 10- to 20-fold. This increased affinity may be the mechanism by which NRP1 enhances VEGFR2 signalling, thereby promoting the angiogenesis of vascular ECs [20].

Our circRNA sequencing data indicated that circGSE1 was significantly downregulated in OECs. To further reveal the role of circGSE1 in angiogenesis, we demonstrated that it functions as a miR-323-5p sponge to regulate angiogenesis via the circGSE1/miR-323-5p/NRP1 axis, thus potentially representing a new therapeutic target for angiogenesis during ageing.

## MATERIALS AND METHODS

### Cell culture

Mouse aortic endothelial cells (MAECs) were obtained from the aortas of male C57BL/6 mice by trypsin digestion. The cells were cultured in DMEM (Thermo Fisher Scientific) supplemented with 10% fetal bovine serum (FBS, Thermo Fisher Scientific) and 1% penicillin/streptomycin in a cell incubator at 37° C and 5% CO_2_. Old mice were 18 months old, while young mice were 4 weeks old. Aortic ECs were obtained from 3 mice in the same batch, and cells were used at passages 3-5 in experimental procedures. All mouse studies were consistent with the Helsinki Declaration and were approved by the Experimental Animal Ethics Committee of the First Affiliated Hospital of Sun Yat-Sen University (Approval NO.2020-399).

### Whole-transcriptome sequencing

The Hi-Seq platform was used for whole-transcriptome sequencing [21]. The sequencing library was constructed by removing rRNAs from the total RNA of 8 samples (MAECs from 4 young and 4 old mice). Clean reads were obtained by removing adapter sequences, N bases, and low-quality fragments. Then, the TopHat programme (v2.0.14) was used to map the fragments to the mouse genome. The DESeq algorithm was used to analyse differentially expressed genes, circular structures were predicted with Accurate CircRNA Finder Suite (ACFS) for circRNA identification. Gene Ontology and pathway analyses were performed to elucidate the biological significances and important pathways of differentially expressed genes. miRanda tools and RNAhybrid algorithms are used to explore competitive endogenous RNA (ceRNA) relationships.

### Cell transfection

CircGSE1 siRNA (RiboBio) and plasmids (Geneseed) was transfected into cells using Lipofectamine 3000 (Thermo Fisher Scientific) in serum-free medium for 6 hours at 37° C cell and 5% CO_2_; thereafter, the medium was replaced with medium supplemented with 10% serum. The transfection concentrations of siRNA and Overexpression plasmids were 50 nmol and 2.5 μg/ml, respectively. RNA was extracted 48 hours after cell transfection, and total protein was extracted 48 to 72 hours after cell transfection for subsequent experiments. The sequences of the siRNA used in this study are shown in Supplementary Table 2.

### RNA extraction and qRT-PCR

Aortic ECs were collected from each group of mice, and total RNA was extracted using TRIzol reagent (Invitrogen). The concentration and purity of RNA were determined by spectrophotometry, and RNA from a single sample was reverse-transcribed into cDNA using a first-strand cDNA synthesis kit (TaKaRa). qRT-PCR was performed using TaKaRa SYBR Green reagent and Roche Light Cycler 480. GAPDH was used as the internal reference gene for circGSE1 and NRP1, and U6 was used as the internal reference gene for miRNA. qRT-PCRs were repeated three times, and differences were calculated by the 2^-ΔΔCt^ method. All primer sequences are listed in Supplementary Table 1.

### RNase R experiment

RNA (10 μg) from MAECs was incubated at 37° C with RNase R (3 U/μg, Geneseed) for 30 minutes. Then, the treated RNA was reverse transcribed using divergent primers or polymeric primers, and nucleic acid electrophoresis was performed after qRT-PCR detection.

### RNA immunoprecipitation

RNA immunoprecipitation (RIP) assay was performed using Magna RIP™ RNA-binding protein immunoprecipitation kit (Millipore). OECs was incubated with magnetic beads conjugated with anti-Argonaute 2 (AGO2) or anti-IgG antibody (Millipore) for 6 h at 4° C. The beads were washed and incubated with Proteinase K to remove proteins. Finally, isolated RNA was extracted using TRIzol Reagent (Takara), then, the purified RNA was subjected to agarose gel electrophoresis and qRT-PCR analysis.

### Nucleic acid electrophoresis

cDNA and gDNA PCR products were detected using 3% agarose gel electrophoresis in TAE buffer solution at 100 V for 45 minutes, and DNA markers were used to identify 100-500bp fragments (Accurate Biology). The gel was illuminated with an ultraviolet detector.

### Fluorescence *in situ* hybridization (FISH)

The localization of circGSE1 and miR-323-5p in the vascular ECs was detected by FISH. The circGSE1 probe labelled with CY3 and the miR-323-5p probe labelled with FITC were designed and synthesized by Geneseed Biology. The circGSE1 and miR-323-5p probes were hybridized overnight according to the manufacturer’s instructions. Images were captured by a Zeiss LSM710 laser scanning confocal microscope (Zeiss Instruments). The immunofluorescence signals of circGSE1 and miR323-5p were quantified with software Fiji setting threshold automatically by Otsu filter. Colocalization values (Pearson, Manders’ coefficients) were calculated from eight representative cell images using the colocalization plugin JACoP (Just another colocalization plugin) [22]. All the experiments were repeated three times. The sequences of circGSE1 and miR-323-5p are listed in Supplementary Table 2.

### Western blotting

WB was used to determine the relative expression levels of NRP1. ECs were incubated under normal culture conditions for 48-72 hours and then frozen in RIPA buffer (Thermo Fisher Scientific). Protein concentrations were determined using the BCA protein assay kit (Thermo Fisher Scientific). Equivalent (20 μg) amounts of protein were separated by 10% SDS-PAGE and transferred to a PVDF membrane (Millipore,). After blocking with TBST supplemented with 5% bovine serum albumin (BSA) (BioFroxx), the PVDF membrane was incubated with anti-rabbit NRP1 primary antibodies at 4° C and then with an anti-rabbit secondary antibody. NovexECL (Invitrogen) was used for detection, and ImageJ software (version 1.8.0) was used for signal quantification. The Primary antibodies are listed in Supplementary Table 3.

### Luciferase assays

CircGSE1 sequences with a mutated miR-323-5p-binding site were obtained using a QuikChange II site-directed mutation kit (Stratagene). Wt-miR-323-5p and mut-miR-323-5p were designed by Giesee. The negative control (NC) or circGSE1 sequence was cotransfected with wt-miR-323-5p or mut-miR-323-5p into HEK-293T cells using Lipofectamine 3000 (Thermo Fisher Scientific). Twenty-four hours after transfection, the luciferase efficiency was measured by a dual-luciferase reporter and detection kit (TransGen Biotech). All the experiments were repeated three times.

### *In vitro* wound healing assay

Transfected cells seeded in 6-well plates were cultured until 90% confluence. A 1-ml sterile pipette tip was used to generate a scratch at a point relative to the reference, after which the cells were washed with sterile PBS and observed under a microscope (Olympus Co., Ltd.) to obtain reference images. Then, the cells were cultured in serum-free medium for 24 hours, and a second set of images of the same area was obtained. ImageJ software was used for the statistical analysis of scratch healing.

### Transwell assay

A chamber (Corning Life Sciences) with a pore diameter of 8 μm was inserted into a 24-well plate. Then, 500 μl of medium containing 10% FBS was added to the lower chamber, and the treated ECs as well as 300 μl of serum-free medium were added to the upper chamber for 24 hours. After the culture period ended, 4% paraformaldehyde for 20 minutes and then attained with crystal violet was used for staining for 15 minutes. The chamber wall was then gently wiped with a cotton ball, and the migrated cells were counted in five random areas using an optical microscope (Olympus Co., Ltd).

### Tube formation experiment

A 24-well plate was precooled at 4° C overnight, filled with 200 μl of Matrigel matrix (Corning Life Sciences), incubated at 4° C for 6 minutes, and placed in a cell culture incubator for 30 minutes. Then, 150000 cells/well were added to the solidified matrix, placed in a cell culture incubator for 6 hours, and imaged using an optical microscope. ImageJ software was used for tube length measurements.

### Cell proliferation evaluation via the EdU assay

ECs were inoculated into a 96-well plate and cultured normally until reaching. 60-70% confluence, after which a c10310-1/-2/-3 kit (RiboBio) was used to assess the proliferation of cultured cells according to the instructions, and a fluorescence microscope (Leica) was used assess their proliferative by measuring the proportion of EdU-positive cells.

### Animal model

C57BL/6 mice (GemPharmatech Co., Ltd) ageing 18 months and 4 weeks were used in the experiment. All procedures were approved by the Animal Ethics Committee of the First Affiliated Hospital of Sun Yat-Sen University, and animal models of unilateral and hindlimb ischemia were established based on previous studies. The left groin area of the mouse was anaesthetized with isoflurane, and a 1-cm oblique incision was made. After the subcutaneous tissue was separated, the femoral artery sheath was opened, the femoral vein and femoral nerve were separated, and the femoral artery was located. The femoral artery was ligated with 7-0 silk thread, and removed, and the incision was sutured. The right hind leg served as a control [23]. Two weeks before the surgery, the mice were divided into four groups as follows: elderly mice injected with AAV9-circGSE1, elderly mice injected with AAV9-NC, elderly mice injected with PBS and young mice injected with PBS. In the two-week before preoperative, AAV9 (30 μl, 10^10^ vg in each leg) and PBS (30 μl, each leg) were injected into the bilateral posterior calf gastrocnemius muscle [24]. The right hind limb was the control. Blood flow was observed pre- and postoperatively at 0, 3, 7, 14, 21 and 28 days using an animal blood flow imaging perfusion apparatus. On the 28th day after the operation, the mice were sacrificed by the intraperitoneal injection of high-dose pentobarbital, and gastrocnemius muscle immunohistochemistry, qPCR, and other experiments were performed.

### Blood flow measurements

Blood flow measurements were performed using a small-animal blood flow imaging perfusion machine. At 0, 3, 7, 14, 21 and 28 days after surgery, blood flow was measured by PeriCamPS I (Perimed) in a constant temperature, humidity and sound insulation environment (room temperature of 24° C, no excessive light and noise). The imaging data were analysed using PIMSoft (Perimed) software [24], and the ratio of ischemic limb blood flow to normal limb blood flow was used as the index of ischemic limb blood flow recovery.

### Immunohistochemistry

Mouse gastrocnemius muscle sections (3 μm) were treated with a hydrogen peroxide blocker and incubated with primary anti-CD31 and anti-NRP1 antibody. After overnight incubation and washing, the muscle sections were incubated with a secondary antibody, exposed to 3,3-diaminobenzidine reagent (DAB; Solarbio), and stained with haematoxylin. The muscle slices were observed and imaged using an Olympus BX51 microscope (Olympus).

### Statistical analysis

All data are presented as the mean ± standard deviation (SD). GraphPad Prism 8.0.1 was used for statistical analysis. Independent sample t-tests were used for comparisons between two groups, and one-way analysis of variance (ANOVA) was used for comparisons among three or more groups.

### Data availability statement

The data that support the results of this study are available from the corresponding author upon reasonable request.

## RESULTS

### CircRNA sequencing identifies differentially expressed circRNAs in endothelial cell aging

**Figure 1:**
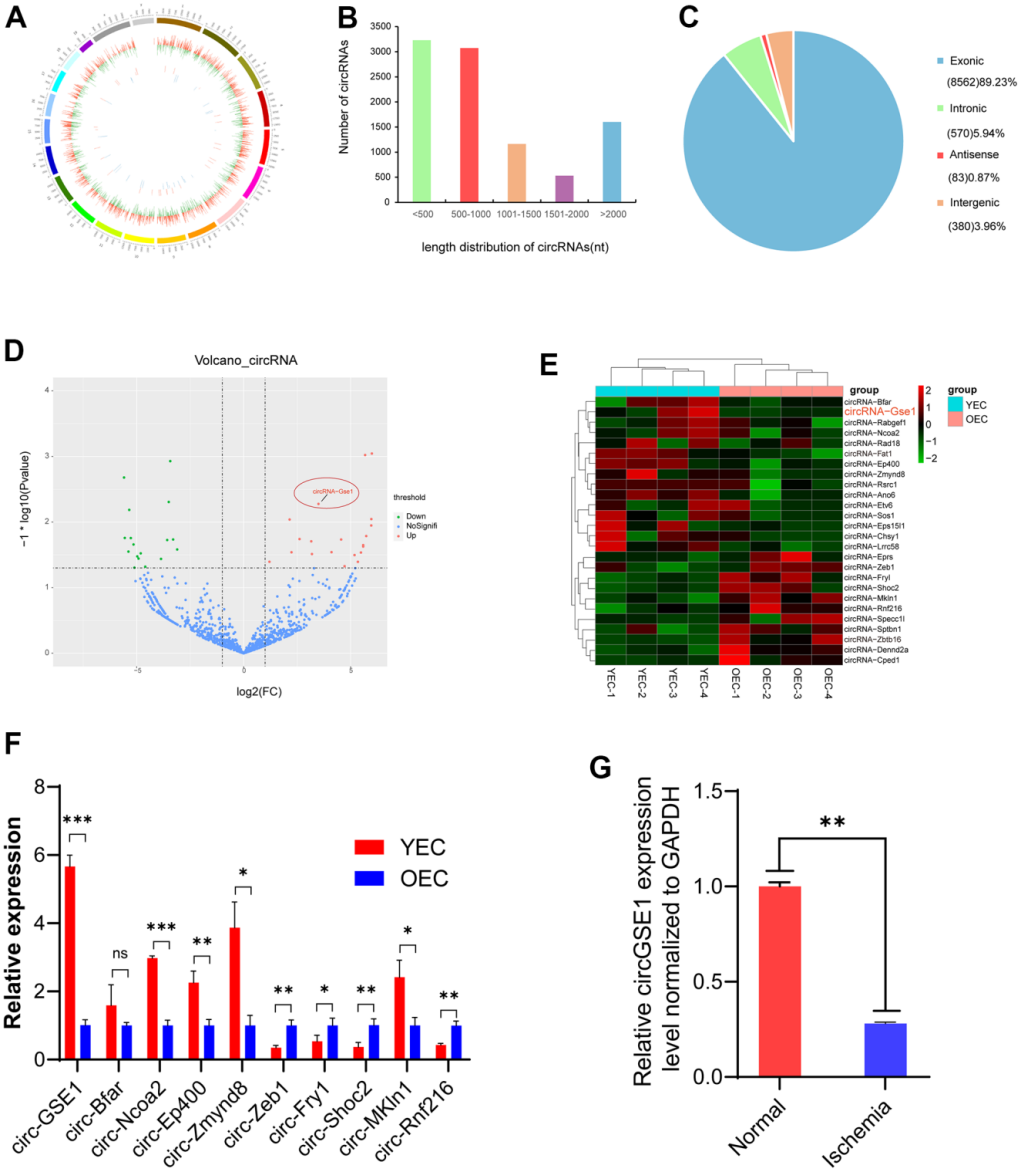
**Profiling of circRNAs in old endothelial cells (OECs) and young endothelial cells (YECs).** (**A**) The circle shows the distribution and expression of differentially expressed circRNAs and mRNAs on mouse chromosomes. The outer, middle and inner layers represent the mouse chromosomes, mRNAs and circRNAs, respectively. Red represents upregulation, and green represents downregulation in YECs. (**B**) The distribution of circRNAs of different lengths in YECs compared with OECs. (**C**) Different mouse genomic sources of differential circRNAs. (**D**) Volcano map of circRNAs in YECs compared with OECs. Red represents upregulation, green represents downregulation, and blue represents no significant difference. (**E**) The heat map shows the differentially expressed circRNAs in OECs and YECs. The red bars represent upregulation, and the blue bars represent downregulation. (**F**) qRT-PCR shows ten of the most significant differences circRNAs expression in YECs compared with OECs; the expression was normalized to that of GAPDH (n=3). (**G**) qRT-PCR analysis of circGSE1 expression in the lower limb gastrocnemius muscles of mice with femoral artery ligation ischaemia; expression was normalized to that of GAPDH (n=3). (The data are expressed as the mean ± SD, *P < 0.05, **P < 0.01 versus the negative control).

To study ageing EC-related angiogenesis, we performed whole-transcriptome sequencing. We identified 26 significantly differentially expressed circRNAs by comparing YEC and OEC samples. The circogram shows the distribution of the detected and significantly expressed circRNAs on mouse chromosomes (Figure 1A). We statistically analysed the length distributions statistics of the detected circRNAs, which indicated that most of the circRNA were less than 1000 nt in length (Figure 1B). Regarding their genomic origins, most of the circRNAs originated from exons (Figure 1C). The volcano plot shows the detected and significantly expressed circRNAs, with red and blue dots representing significantly up- and downregulated circRNAs in YECs compared with OECs, respectively (Figure 1D).

### CircGSE1 is significantly downregulated in MAECs of ageing mice and ischemic muscles

The heat map shows differentially expressed circRNAs in OECs and YECs, and circGSE1 was significantly upregulated in YECs compared with OECs (Figure 1E). We used qRT-PCR to verify the 10 most significantly different circRNAs (5 upregulated and 5 downregulated), which were basically consistent with the sequencing results. And we found circGSE1 was the most significantly upregulated in YECs (Figure 1F). Meanwhile, we found that circGSE1 was expressed at significantly lower levels in the ischemic gastrocnemius muscle samples than that in control samples (Figure 1G).

### CircGSE1 characteristics and differences in organization

**Figure 2:**
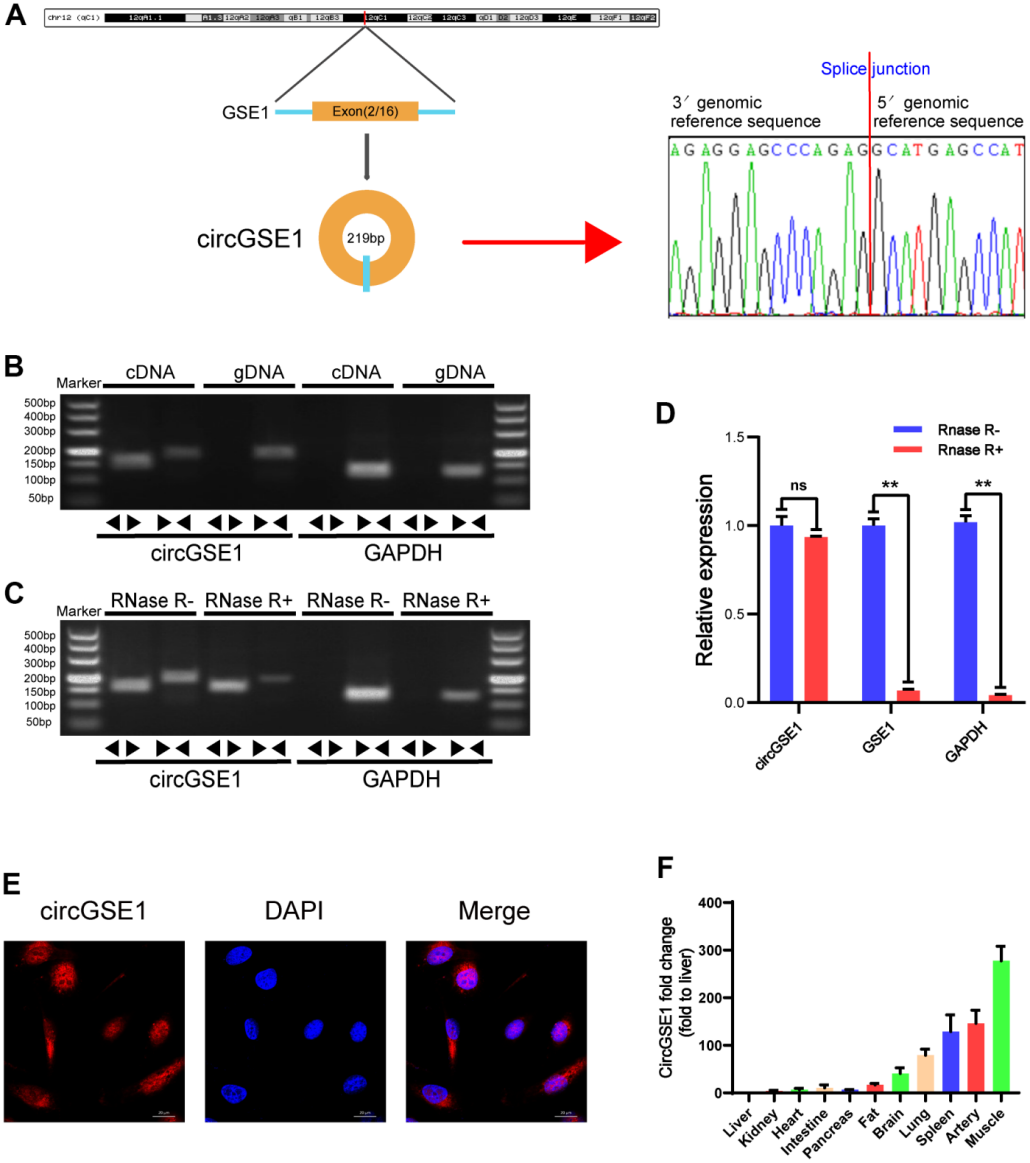
**Validation of circGSE1 and differences in organization.** (**A**) Sanger sequencing of the band amplified with divergent primers confirms head-to-tail splicing of circGSE1. (**B**) Agarose gel electrophoresis validated the expression of circGSE1 in MAECs. CircGSE1 was amplified by divergent primers in cDNA but not gDNA, and GAPDH was used as a negative control. (**C**, **D**) In the presence or absence of RNase R, the expression of circGSE1 and linear GSE1 RNA in MAECs was detected by qRT-PCR, and nucleic acid electrophoresis or qRT-PCR was then performed. (**E**) FISH experiments proved that most circGSE1 is localized in the cytoplasm. Nuclei were stained blue, and cytoplasmic circGSE1 was stained red. (magnification, 400×, scale bar, 20 μm). (**F**) The circGSE1 expression levels in different mouse organs were analysed by qRT-PCR, revealing higher expression in muscle, artery and splenic organs. (The data are expressed as the mean ± SD, *P < 0.05, **P < 0.01 versus the negative control).

Our study confirmed the head-to-tail splicing of circGSE1 in the circGSE1 PCR product by Sanger sequencing and determined its genome size and sequence, as reported by the circBase database (Figure 2A). As a result, we also designed convergent primers for GSE1 RNA and specific divergent primers for the amplification of GSE1. cDNA and gDNA were extracted from OECs and analysed by PCR and agarose gel electrophoresis, revealing that circGSE1 was only amplified from cDNA, and not from gDNA (Figure 2B). We also employed RNase R to confirm the stability of circGSE1 using specially designed divergent and convergent primers for PCR and agarose gel electrophoresis. CircGSE1 resisted digestion by RNase R, while linear GSE1 and GAPDH were sensitive to RNase R. In addition, qRT-PCR was used to quantify the expression levels of the back spliced and typical forms of GSE1 with or without RNase R in the cDNA and gDNA of OECs (Figure 2C, 2D). FISH experiments showed that circGSE1 was mostly located in the cytoplasm (Figure 2E). We detected the relative expression of circGSE1 in different mouse organs by qRT-PCR, revealing that circGSE1 expression was higher in muscle, artery and splenic tissues than that in other organs (Figure 2F).

### CircGSE1 promotes the proliferation, migration and tube formation of MAECs *in vitro*

**Figure 3:**
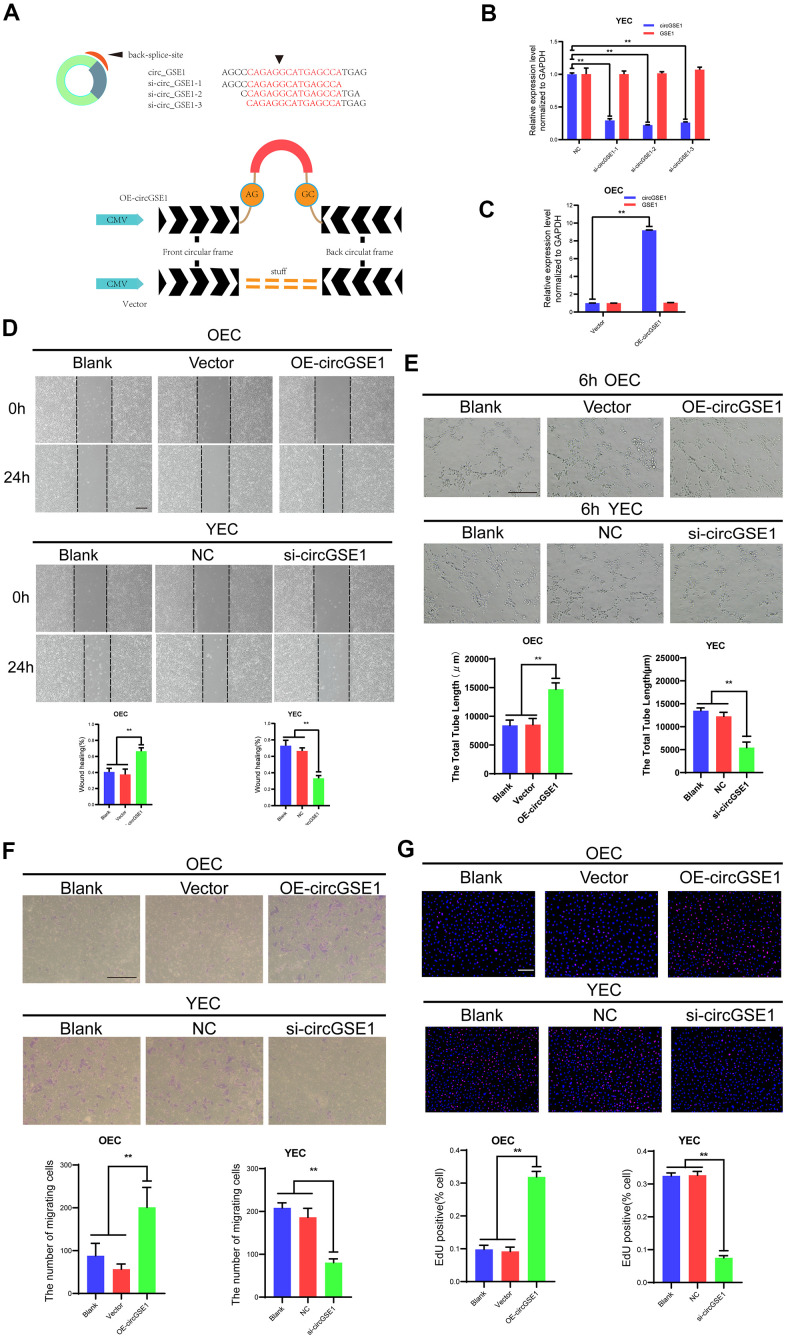
**CircGSE1 promotes the proliferation, migration and tube formation of MAECs *in vitro*.** (**A**) Schematic diagram of the circGSE1 siRNA and overexpression vectors. (**B**) qRT-PCR detection of YECs demonstrating successful knockdown of circGSE1; however, the linear GSE1 RNA expression was not altered significantly (n=3). (**C**) qRT-PCR detection demonstrating successful circGSE1 overexpression in OECs, while the linear GSE1 RNA expression was not changed significantly (n=3). (**D**) Wound healing assay of OECs treated with vector and OE-circGSE1, YECs treated with NC and si-circGSE1 (n=3, scale bar, 200 μm). (**E**) Tube formation assay of OECs treated with vector and OE-circGSE1, YECs treated with NC and si-circGSE1 (n=3, scale bar, 200 μm). (**F**) Migration assay of OECs treated with vector and OE-circGSE1, YECs treated with NC and si-circGSE1 (n=3, scale bar, 200 μm). (**G**) EdU assay of OECs treated with vector and OE-circGSE1, YECs treated with NC and si-circGSE1 (n=3, scale bar, 200 μm). (n=5, scale bar, 100 μm). (The data are expressed as the mean ± SD, *P < 0.05, **P < 0.01 versus the negative control).

To explore the function of circGSE1 in MAECs, we designed three siRNAs according to the splice site of circGSE1 to inhibit its expression and designed a circGSE1 overexpression vector (Figure 3A). We used qRT-PCR assay to detect the interference effect and found that all three siRNAs could knock down circGSE1, while GSE1 mRNA did not change. We chose to utilize si-circGSE1-2 for subsequent experiments because it had the strongest inhibitory effect (Figure 3B, 3C).

We also conducted a variety of functional experiments to explore the role of circGSE1 in angiogenesis. The wound healing assay showed that compared with the empty vector overexpression and blank control groups, the circGSE1 overexpression group exhibited an enhanced OEC migration ability, while the YEC migration in the circGSE1-knockdown group was inhibited compared with that in the siRNA-NC and the blank control groups, (Figure 3D). The tube formation assay demonstrated that compared with that in the empty vector overexpression and blank control groups, the OEC tube formation ability in the circGSE1 overexpression group was enhanced, while that of YECs in the circGSE1-knockdown group was inhibited compared with that in siRNA-NC and blank control groups (Figure 3E). The migration assay showed that compared with that in the empty vector overexpression and blank control groups, the OEC migration ability in the circGSE1 overexpression group was enhanced, while that of YECs in the circGSE1-knockdown group was inhibited compared with that in the siRNA-NC and blank control groups (Figure 3F). The EdU assay indicated that compared with that in the empty vector overexpression and blank control groups, the OEC proliferation in the circGSE1 overexpression group was enhanced, while that of YECs was inhibited, in the circGSE1- knockdown group compared with that in the siRNA-NC and blank control groups (Figure 3G). These functional experiments show that circGSE1 can promote the proliferation, migration and tube formation of MAECs, thereby promoting angiogenesis.

### miR-323-5p is the downstream target of circGSE1

**Figure 4:**
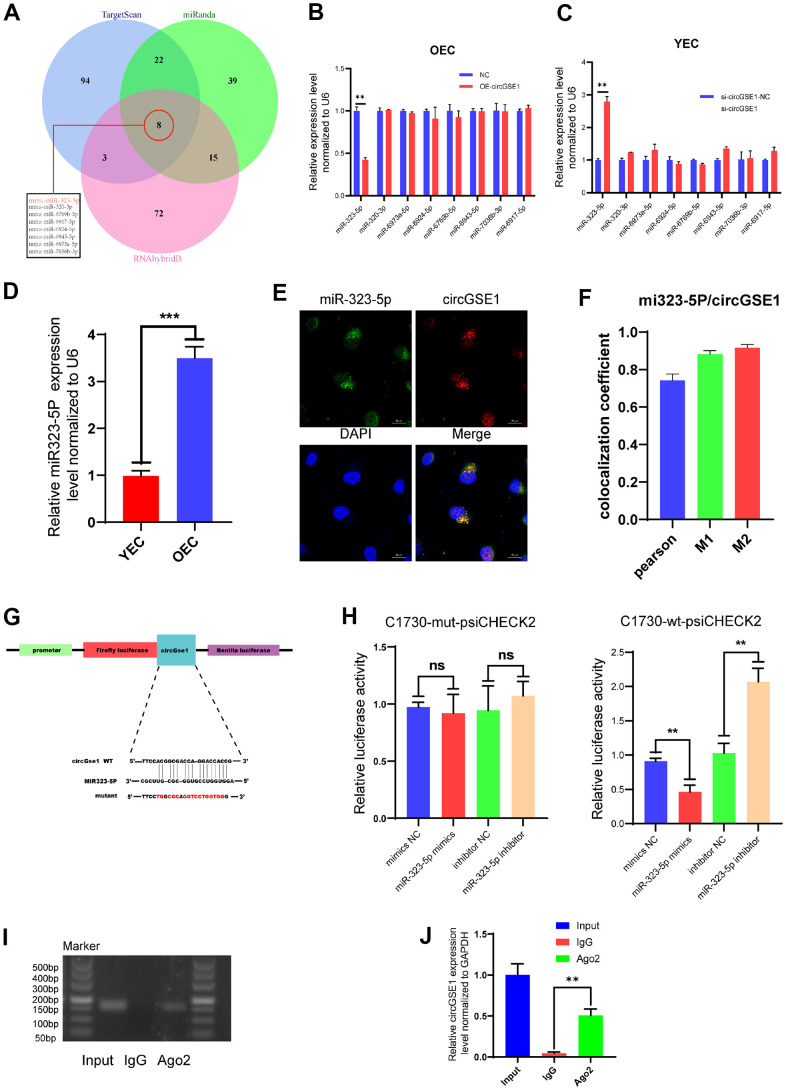
**miR323-5p is the downstream target of circGSE1.** (**A**) The downstream miRNA targets of circGSE1 as predicted by the TargetScan, miRanda and RNAhybridB databases. (**B**) The relative OEC expression of the downstream miRNA target of circGSE1 predicted by the database as determined by qRT-PCR. (**C**) The relative YEC expression of the downstream miRNA target of circGSE1 predicted by the database as determined by qRT-PCR. (**D**)The relative of miR323-5p expression in YEC and OEC. (**E**, **F**) The FISH experiment showed that most circGSE1 (red) and miR-323-5p (green) are colocalized in the cytoplasm (magnification, 400×, scale bar, 20 μm) (n=3, analysed 8 cells). (**G**) Schematic diagrams of the circGSE1-WT and circGSE1-mutant dual-luciferase reporter vectors. (**H**) Dual-luciferase reporter assays showed that the circGSE1 wt 3’UTR but not the mut 3’UTR was downregulated by miR-323-5p mimics or upregulated by the miR-323-5p inhibitor. (**I**, **J**) The Anti-Ago2 RIP assay was executed in OEC, followed by nucleic acid electrophoresis and qRT-PCR to detect circGSE1. (The data are expressed as the mean ± SD, *P < 0.05, **P < 0.01 versus the negative control).

To further explore the specific mechanism by which circGSE1 promotes angiogenesis, we used three databases (TargetScan, miRanda and RNAhybrid) to predict the downstream microRNA targets of circGSE1. The prediction results showed that 8 microRNAs are potentially downstream targets of circGSE1, miR-323-5p, miR-320-3p, miR-6973a-5p, miR-6924-5p, miR-6769b-5p, miR-6943-5p, miR-7036b-3p, and miR-6917-5p (Figure 4A). We used qRT-PCR to quantify the relative expression levels of the 8 predicted microRNAs in OECs overexpressing circGSE1 and in YECs after siRNA-mediated knockdown of circGSE1, and only miR-323-5p was differentially expressed (Figure 4B, 4C). We also found that miR323-5p was upregulated in OECs compared with YECs (Figure 4D). Meanwhile, considering that circRNAs could serve as miRNA sponges in the cytoplasm, FISH experiments showed that circGSE1 and miR-323-5p were mostly colocalized in the cytoplasm (Figure 4E, 4F). In addition, bioinformatic analysis was used to identify the portion of the circGSE1 sequence that may bind to miR-323-5p (Figure 4G).

To verify the binding of circGSE1 and miR-323-5p, we synthesized circGSE1-wt-psiCHECK2 and circGSE1-mut-psiCHECK2 dual-luciferase assay vectors. Herein, circGSE1 wt 3’UTR but not the mut 3’UTR was downregulated by miR-323-5p mimics or upregulated by the miR-323-5p inhibitor, which suggested that a direct interaction occurs between circGSE1 and miR-323-5p (Figure 4H). It was widely acknowledged that miRNAs regulated target mRNA expression by binding to Argonaute 2 (Ago2), an essential component of RNA-induced silencing complex (RISC). Subsequently, RNA immunoprecipitation (RIP) assays were performed in OECs to pull down the RNA transcripts that bound to Ago2. Results of agarose gel electrophoresis and qRT-PCR assays indicated that circGSE1 was pulled down by anti-Ago2 as compared with those in the input control (Figure 4I, 4J).

### MiR323-5p inhibits the proliferation, migration and tube formation of MAECs

**Figure 5:**
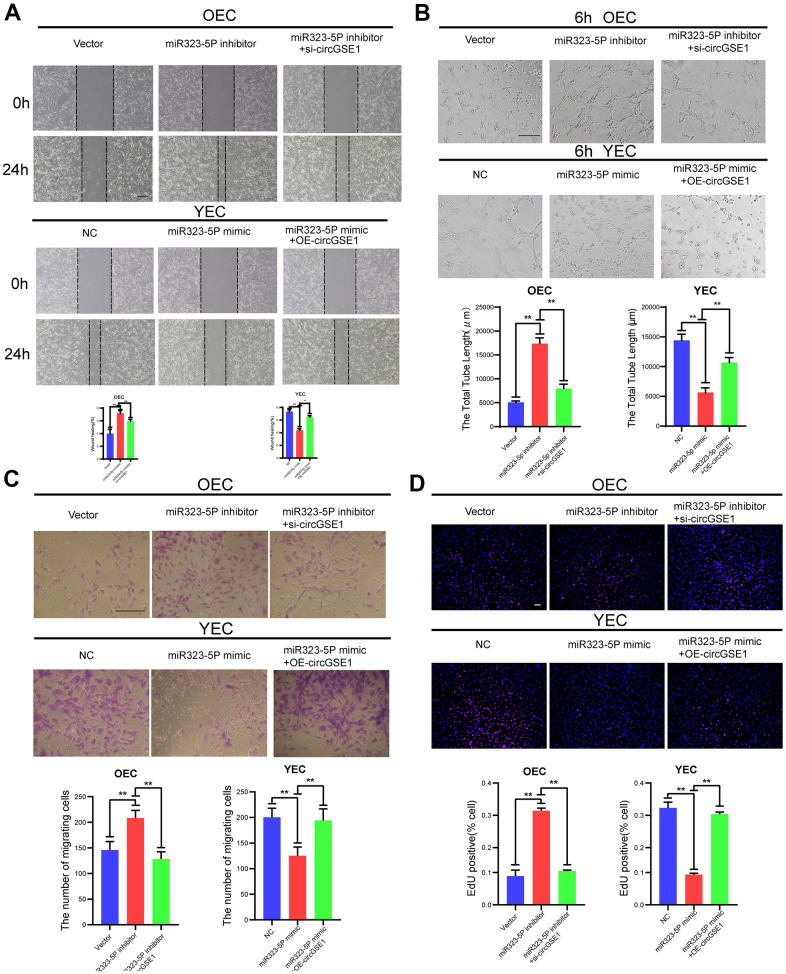
**CircGSE1 regulates the angiogenic ability of MAECs via the miR-323-5p.** (**A**) Wound healing assay of OECs treated with vector, the miR-323-5p inhibitor or the miR-323-5p inhibitor + circGSE1 siRNA and YECs treated with the NC, miR-323-5p mimic or miR-323-5p mimic+OE-circGSE1. The graph indicates the quantification of the wounded area 24 hours after scratching (n=3, scale bar: 200 μm). (**B**) Tube formation assay of OECs treated with the vector, miR-323-5p inhibitor or miR-323-5p inhibitor+circGSE1 siRNA and YECs treated with the NC, miR-323-5p mimic or miR-323-5p mimic+OE-circGSE1 (n=3, scale bar, 200 μm). (**C**) Migration assay of OECs treated with the vector, miR-323-5p inhibitor or miR-323-5p inhibitor + circGSE1 siRNA and YECs treated with the NC, miR-323-5p mimic or miR-323-5p mimic+OE-circGSE1 (n=5, scale bar, 200 μm). (**D**) EdU assay of OECs treated with the vector, miR-323-5p inhibitor or miR-323-5p inhibitor + circGSE1 siRNA and YECs treated with the NC, miR-323-5p mimic or miR-323-5p mimic+OE-circGSE1 (n=5, scale bar, 200 μm). (The data are expressed as the mean ± SD, *P < 0.05 **P < 0.01 versus the negative control).

To further study the interaction among circGSE1 and miR-323-5p, we conducted a series of functional experiments. Wound healing experiments showed that the miR-323-5p inhibitor enhanced the migration ability of OECs, but siRNA targeting circGSE1 suppressed this effect. Moreover, the miR-323-5p mimic decreased the migration ability of YECs, but this inhibitory effect was reversed by co-transfection of OE-circGSE1 (Figure 5A). Tube formation experiments showed that the miR-323-5p inhibitor enhanced the tube formation ability of OECs, while the siRNA targeting circGSE1 suppressed this effect. Moreover, miR-323-5p mimics decreased the tube formation ability of YECs, but this inhibitory effect was reversed by co-transfection of OE-circGSE1 (Figure 5B). Migration experiments showed that inhibiting miR-323-5p enhanced the migration ability of OECs, but co-transfection of the miR-323-5p inhibitor with the siRNA targeting circGSE1, decreased their migration ability. Overexpressing miR-323-5p via mimics decreased the migration ability of YECs, while co-transfection with OE-circGSE1 increased their migration ability (Figure 5C). EdU experiments showed that inhibiting miR-323-5p enhanced the proliferation of OECs, while co-transfection of the miR323-5p inhibitor with siRNA targeting circGSE1 decreased the proliferation of OECs. Overexpressing miR-323-5p via mimics decreased the proliferation ability of YECs, but co-transfection with OE-circGSE1 increased their proliferation (Figure 5D).

### NRP1 is the downstream target of miR-323-5p

To continue searching for the downstream targets of circGSE1 and miR-323-5p and explore the specific mechanisms by which they promote angiogenesis, we used three databases (TargetScan, RNAhybrid and Miranda) to jointly predict the downstream targets of miR-323-5p, found 85 mRNAs possibly to be downstream of miR323-5p. Through bioinformatics, it was found that NRP1 had a strong binding with miR323-5p. At the same time, this protein has been reported to play a role in angiogenesis [25–28] and was thus selected for further experiments.

**Figure 6:**
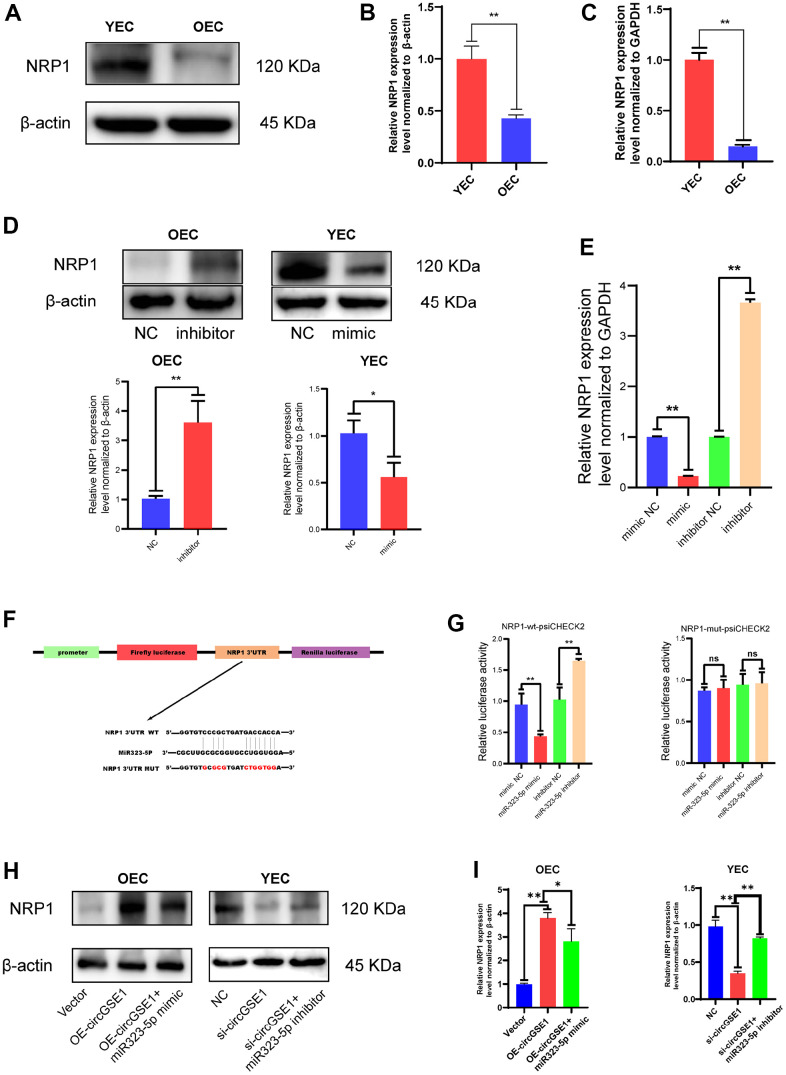
**NRP1 is the downstream target of miR323-5p.** (**A**, **B**) Western blot analysis of the NRP1 expression in OECs and YECs. (**C**) qRT-PCR analysis of the NRP1 mRNA expression in OECs and YECs. (**D**) Western blot analysis of the NRP1 expression in OECs transfected with negative control and miR323-5p inhibitor, YECs transfected with negative control and miR323-5p mimics. (**E**) qRT-PCR analysis of the NRP1 mRNA expression in YECs transfected with negative control and miR323-5p mimics, OECs transfected with negative control and miR323-5p inhibitor. (**F**) Schematic diagrams of the circGSE1-WT and circGSE1-mutant dual-luciferase reporter vectors. (**G**) Dual-luciferase reporter assays showed that the circGSE1 wt 3’UTR but not the mut 3’UTR was downregulated by miR-323-5p mimics or upregulated by the miR-323-5p inhibitor. (**H**, **I**) WB analyses of OECs treated with the vector, OE-circGSE1 or OE-circGSE1 + miR323-5p mimic and YECs treated with the NC, si-circGSE1 or si-circGSE1+miR323-5p inhibitor. (The data are expressed as the mean ± SD, *P < 0.05, **P < 0.01 versus the negative control).

Through WB assay and PCR assay, we found that NRP1 expression was up-regulated in YEC compared with OEC (Figure 6A, 6B). The expression of NRP1 was increased after transfection of a miR-323-5p inhibitor into OECs, and its expression was decreased after transfection of miR-323-5p mimics into YECs, indicating that miR-323-5p can regulate the expression of NRP1 in ECs (Figure 5D). In addition, bioinformatic analysis revealed the sequence of miR-323-5p that may bind to NRP1 (Figure 6F). To verify the direct binding of miR-323-5p to NRP1, we synthesized NRP1-wt-psiCHECK2 and NRP1-mut-psiCHECK2 dual-luciferase reporter gene vectors. The NRP1 wt 3’UTR but not the mut 3’UTR was downregulated by the miR-323-5p mimics and upregulated by the miR-323-5p inhibitor, which indirectly proved that circGSE1 bind to miR-323-5p (Figure 6G). Then, qRT-PCR and western blot assays demonstrated that overexpression of circGSE1 significantly counteract the mRNA and protein level of NRP1, whereas the co-transfection of si-circGSE1 and miR323-5p inhibitor might eliminate effects in YECs. Similarly, it was also found that the knockdown of circGSE1 significantly decreased the mRNA and protein level of NRP1, whereas co-transfection of OE-circGSE1 and miR323-5p mimics might increase these effects in YECs (Figure 6H, 6I). In conclusion, these data demonstrated that circGSE1 acts as a sponge for miR-323-5p, thus enhancing NRP1expression in MAECs.

### CircGSE1 regulates the angiogenic ability of mouse ECs via the miR-323-5p/NRP1 axis

**Figure 7:**
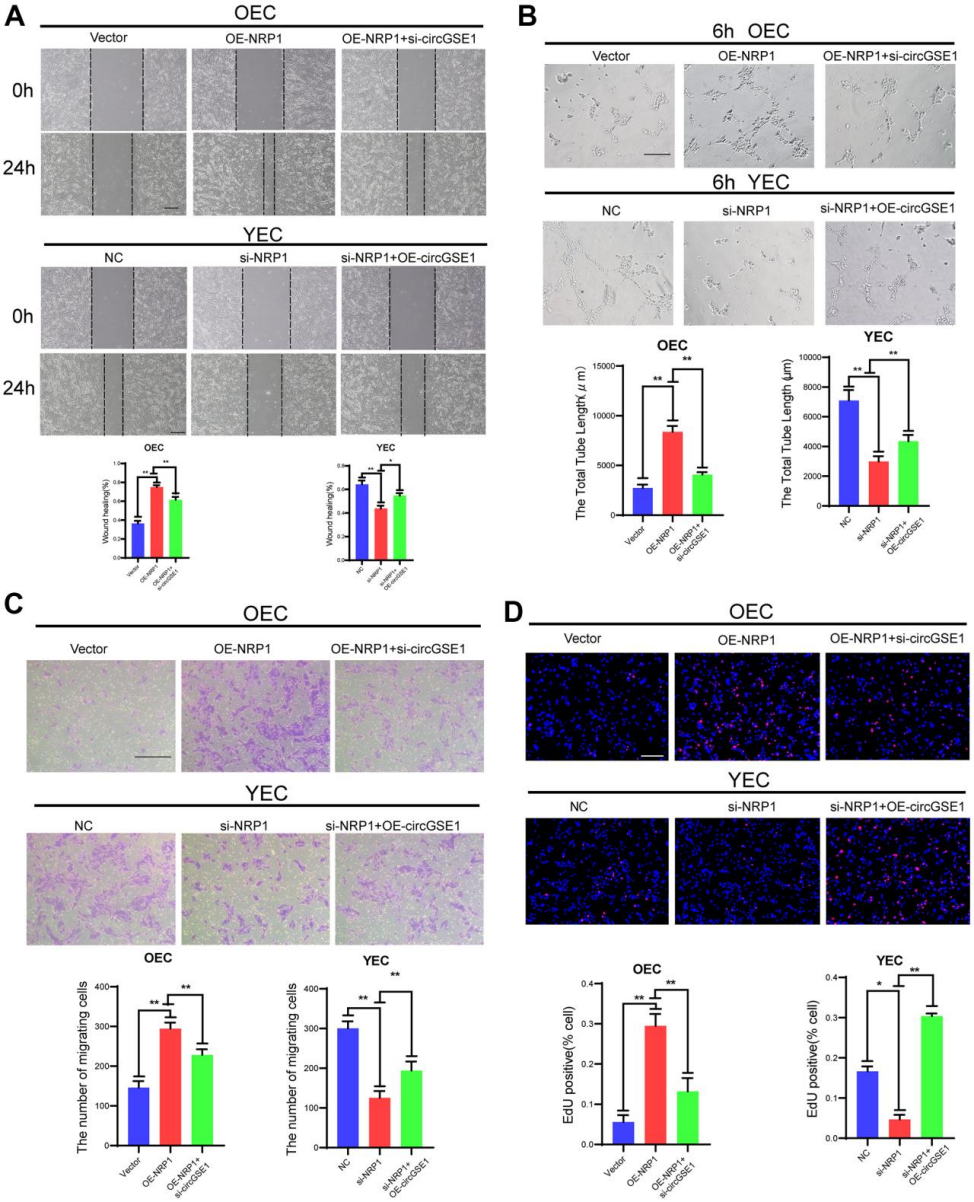
**CircGSE1 regulates the angiogenic ability of MAECs via the NRP1.** (**A**) Wound healing assay of OECs treated with vector, the OE-NRP1 or the OE-NRP1 + circGSE1 siRNA and YECs treated with the NC, NRP1-siRNA or NRP1-siRNA +OE-circGSE1. The graph indicates the quantification of the wounded area 24 hours after scratching (n=3, scale bar: 200 μm). (**B**) Tube formation assay of OECs treated with the vector, the OE-NRP1 or the OE-NRP1 + circGSE1 siRNA and YECs treated with the NC, NRP1-siRNA or NRP1-siRNA +OE-circGSE1 (n=3, scale bar, 200 μm). (**C**) Migration assay of OECs treated with the vector, the OE-NRP1 or the OE-NRP1 + circGSE1 siRNA and YECs treated with the NC, NRP1-siRNA or NRP1-siRNA +OE-circGSE1 (n=5, scale bar, 200 μm). (**D**) EdU assay of OECs treated with the vector, the OE-NRP1 or the OE-NRP1 + circGSE1 siRNA and YECs treated with the NC, NRP1-siRNA or NRP1-siRNA +OE-circGSE1 (n=5, scale bar, 200 μm). (The data are expressed as the mean ± SD, *P < 0.05 **P < 0.01 versus the negative control).

To further study the interaction among circGSE1, miR-323-5p and NRP1, we conducted a series of functional experiments. Wound healing experiments showed that the OE-NRP1 enhanced the migration ability of OECs, but siRNA targeting circGSE1 suppressed this effect. Moreover, the siRNA targeting NRP1 decreased the migration ability of YECs, but this inhibitory effect was reversed by co-transfection of OE-circGSE1 (Figure 7A). Tube formation experiments showed that the OE-NRP1 enhanced the migration ability of OECs, but siRNA targeting circGSE1 suppressed this effect. Moreover, the siRNA targeting NRP1 decreased the migration ability of YECs, but this inhibitory effect was reversed by co-transfection of OE-circGSE1 (Figure 7B). Migration experiments showed that overexpressing NRP1 enhanced the migration ability of OECs, but co-transfection of the OE-NRP1 plasmid with the siRNA targeting circGSE1, decreased their migration ability. Inhibiting NRP1 via siRNA decreased the migration ability of YECs, while co-transfection with OE-circGSE1 increased their migration ability (Figure 7C). EdU experiments showed that overexpression NRP1 enhanced the proliferation of OECs, while co-transfection of the siRNA targeting circGSE1 decreased the proliferation of OECs. Inhibiting circGSE1via siRNA decreased the proliferation ability of YECs, but co-transfection with OE-circGSE1 increased their proliferation (Figure 7D).

### CircGSE1 can promote blood flow recovery and angiogenesis in the ischemic limbs of ageing mice

**Figure 8:**
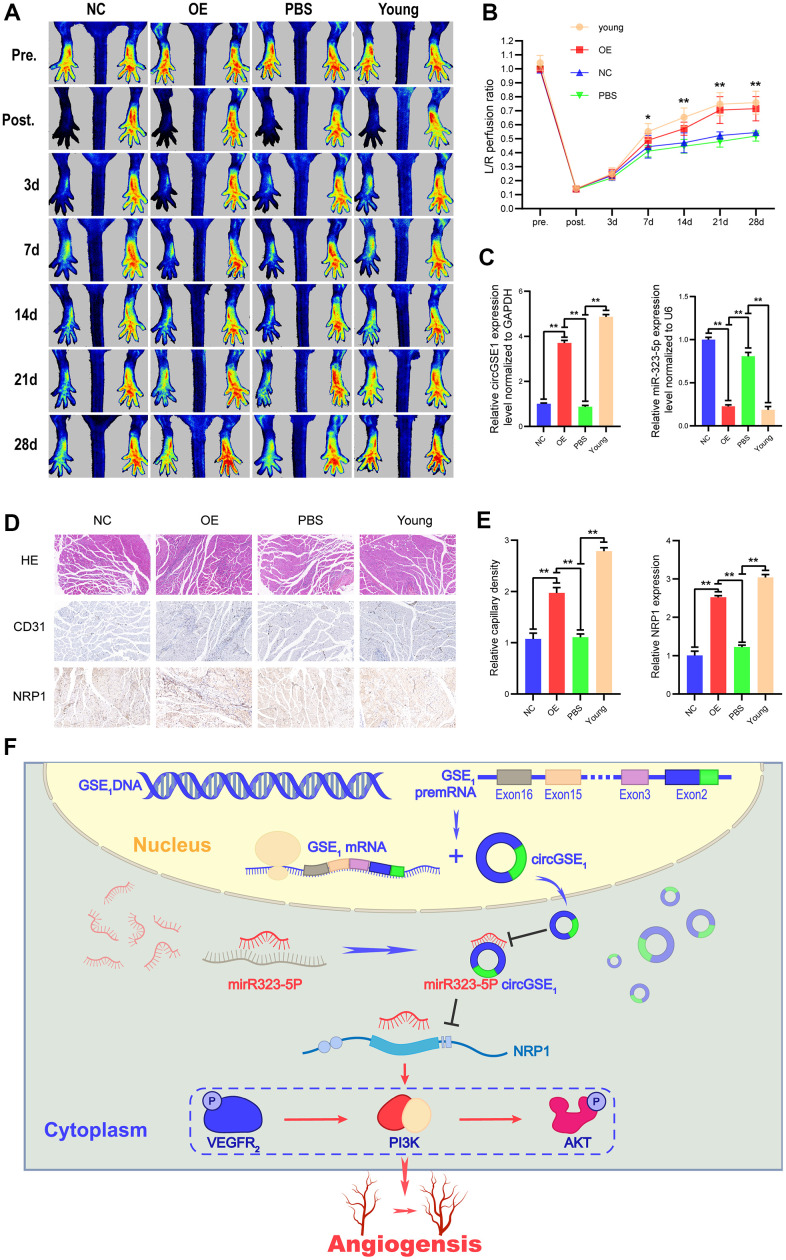
**Overexpression of circGSE1 can promote blood flow recovery and angiogenesis in ageing mice.** (**A**) Imaging of blood flow through the mouse femoral artery 3, 7, 14, 21, and 28 days after surgery (n=5). (**B**) The blood flow through the mouse ischaemic limbs was detected, and the results are expressed as the ratio of the perfusion of the left limb (ischaemic) to that of the right limb (control, nonischaemic) on the 7th, 14th, and 21st days after the surgery (n=5). (**C**) qRT-PCR analysis of circGSE1 and mi323-5p expression in the ischaemic left lower limb gastrocnemius muscles in the young mouse group, old mouse circGSE1 overexpression group and old mouse PBS group compared to the old mouse NC group; expression levels were normalized to that of GAPDH. (**D**, **E**) Representative images of HE, anti-CD31 and anti-NRP1 immunohistochemical staining of mouse left lower limb gastrocnemius muscles. The CD31 and NRP1 expression levels in the young mouse group, old mouse circGSE1 overexpression group and old mouse PBS group were compared to those in the old mouse NC group (n=5, scale bar, 100 μm) (The data are expressed as the mean ± SD, *P < 0.05, **P < 0.01). (**F**) Schematic diagram of the mechanism by which circGSE1 promotes angiogenesis in ageing mice.

To further study the role of circGSE1, we successfully established acute lower limb ischemia models in ageing mice (C57BL/6, male,18 months old) and young mice (C57BL/6, male,4 weeks old). The ageing mice were randomly divided into 3 groups (PBS, negative control (NC), and overexpression (OE)), and a young mouse group were included (see the methods section for details). On 7th, 14th, 21st, and 28th days after the operation, the blood flow recovery in the lower limbs of the young mouse group and the OE-circGSE1 ageing mouse group was better than that in the NC ageing mouse group and PBS group, demonstrating that circGSE1 can promote angiogenesis in the ischemic lower limbs of ageing mice (Figure 8A, 8B). We also used qRT-PCR to verify the expression of circGSE1 and miR-323-5p in the ischemic lower limbs gastrocnemius muscles of mice in the different groups, revealing that circGSE1 was overexpressed in young and old mice of the circGSE1 group. The level of miR-323-5p in the OE-circGSE1 group was significantly higher than that in the NC and PBS groups in ageing mice, while miR-323-5p was expressed at significantly lower level in the young mouse and OE-circGSE1 ageing mouse group than in the NC and PBS ageing mouse group (Figure 8C). To assess the pathological changes in the ischemic tissue, we prepared tissue sections of the ischemic lower limb gastrocnemius muscles of different groups of mice 28 days after the operation and stained them with anti-CD31 and anti-NRP1. The expression levels of CD31 and NRP1 in the young mouse and circGSE1 OE groups were significantly higher than those in the NC and PBS groups of ageing mice (Figure 8D, 8E).

The above experiments proved that the circGSE1/miR-323-5p/NRP1 axis exists in MAECs. Overall, these results suggest that circGSE1 acts as a miR-323-5p sponge to promote NRP1 expression and thus promote angiogenesis (Figure 8F).

## DISCUSSION

CircRNAs are a unique noncoding RNAs that do not have a 5’ cap or a 3’ poly(A) tail, form a circular structure with covalent bonds, are characterized by endogenous expression, high abundance, conservation and stability [29–31]. Many studies have shown that circRNAs play an important role in the pathogenesis of atherosclerosis, coronary heart disease, hypertension and peripheral arterial ischemic disease [7, 8, 11, 32, 33], and ageing is an important factor that can promote the occurrence and development of cardiovascular-related diseases via ECs [34–36]. However, how circRNAs regulate the senescence of vascular ECs has not yet been reported. Therefore, we herein explored the differentially expressed circRNAs in the aortic ECs of ageing and young mice and studied their effect on ageing-related vascular EC angiogenesis.

Our circRNA sequencing results showed that circGSE1 was downregulated in OECs but upregulated in YECs. We extracted total RNA from the primary aortic ECs of ageing and young mice and verified circRNA expression by qRT-PCR, which was consistent with the sequencing results. In addition, RNase R experiments further verified the stability of circGSE1. We herein found that overexpression of circGSE1 could promote the proliferation, migration and tube formation in OECs, while knocking down circGSE1 attenuated these abilities in YECs. These studies showed that circGSE1 can promote angiogenesis *in vitro*.

Many studies have reported that circRNAs can act as sponges of microRNAs, RNA-binding proteins, and nuclear transcription regulators or can be directly translated into proteins to regulate cell functions [10, 13–15]. In this study, FISH experiments indicated that circGSE1 is mainly located in the cytoplasm. Therefore, we speculate that circGSE1 regulates EC angiogenesis by acting as a microRNA sponge. Fan et al. also found that circleGSE1 promotes cervical cancer cell migration and invasion by acting as miR138-5p sponge [37].

An increasing number of studies have reported that circRNAs can act as sponges for microRNAs and inhibit microRNA activity in mammals [13, 38]. The circRNA CIRS-7 contains 73 conserved miR-7 binding sites and can regulate the miR-7 activity in the central nervous system by acting as a sponge of miR-7 [39]. In cardiovascular disease research, an increasing number of studies have reported the sponge mechanism of circRNAs [8]. CircRNA-0044073 is upregulated in atherosclerosis and promotes the proliferation and invasion of vascular smooth muscle cells by acting as a sponge for miR-107 [40]. CircRNA-0003204 inhibits the proliferation, migration and tube formation of ECs in atherosclerosis through the miR-370-3p/TGFβR2/p-SMAD3 axis [33]. However, the sponge mechanism of circRNAs has not been clearly reported in EC senescence. Our study is the first report that circGSE1 regulates vascular EC senescence via a sponge mechanism. By searching the TargetScan, miRanda, and RNAhybrid databases, we found 8 microRNAs that are potential targets of circGSE1. We assessed the predicted microRNA targets after overexpression of circGSE1 in OECs and knockdown of circGSE1 in YECs. Based on qRT-PCR experimental results, we finally selected miR-323-5p for using in further research, as it was significantly differentially expressed.

MiR-323-5p can reportedly act as a tumour suppressor in human glioma cells by targeting the insulin-like growth factor 1 receptor, can inhibit cell proliferation by blocking IGF-1R-mediated migration [41]; however; its role in ageing-related angiogenesis has not been reported. In this study, miR-323-5p was downregulated after overexpression of circGSE1 in OECs and upregulated after circGSE1 knock down in YECs. Knockdown of miR-323-5p promoted the proliferation, migration and tube formation of OECs, while overexpression of miR-323-5p decreased the proliferation, migration and tube formation abilities of YECs. These studies demonstrate that miR-323-5p can inhibit the angiogenesis of MAECs. The dual-luciferase assay also showed that miR-323-5p is a downstream target of circGSE1, which is consistent with previous predictions. FISH experiments confirmed that miR-323-5p and circGSE1 are mainly colocalized in the cytoplasm. Together, these results show that the circGSE1/miR-323-5p signaling pathway exists in MAECs and regulates the angiogenesis of MAECs.

To further explore the relationship between miR-323-5p and downstream proteins, we searched the miRanda, RNAhybrid and TargetScan databases found NRP1 as a potential target, and this protein can reportedly promote EC angiogenesis through the VEGFA/VEGFR-2 signalling pathway [25–28]. Our circRNA sequencing results showed that the mRNA expression of NRP1 in OECs was downregulated, and its expression in YECs was upregulated. qRT-PCR and WB analyses of miR-323-5p-overexpressing YECs showed that NRP1 expression was downregulated, while these analyses of miR-323-5p-knockdown OECs showed upregulated NRP1 expression. In addition, dual-luciferase assays demonstrated that NRP1 is a direct target of miR-323-5p.

To more clearly study the role of circGSE1 in the angiogenesis of MAECs, we used a miR-323-5p inhibitor in OECs, which enhanced their migration, tube formation and proliferation abilities. However, co-transfection of OECs with both the miR-323-5p inhibitor and si-RNA targeting circGSE1 did not completely ameliorate the promotional effect of miR-323-5p upregulation on OECs. In addition, we used miR-323-5p mimics in YECs, which inhibited their migration, tube formation and proliferation abilities. However, co-transfection of YECs with both the miR-323-5p mimics and OE-circGSE1 did not completely reverse the negative impact of miR-323-5p downregulation on YECs. Together, these results show that circGSE1 can regulate EC angiogenesis in mice by acting as a miR-323-5p sponge.

To investigate the role of circGSE1 in angiogenesis *in vivo*, we established an old C57bcl/6 mouse lower limb ischemia model by ligating and cutting the femoral arteries of the left lower limbs of the mice. After injection of the AAV9-circGSE1 virus into the gastrocnemius muscles of the mice, the blood perfusion recovery of the ischemic limb and the density of new capillary vessels in the gastrocnemius muscle were better than those in the control group of old mice but slightly weaker than those in the young mouse group. These results suggest that an increase in circGSE1 levels *in vivo* can promote the recovery of blood perfusion and the regeneration of new capillaries in the ischemic lower limbs of ageing mice.

This study has some limitations, more research is needed to confirm how age affects circGSE1 expression, whether it affects angiogenesis through other targets, and whether miR-323-5p affects the NRP1 mechanism of action.

In summary, we clarified the important role of circGSE1 in regulating angiogenesis through the miR-323-5p/NRP1 axis *in vitro* and *in vivo*. This study shows that increasing circGSE1expression can promote the regeneration of senescent blood vessels through the miR-323-5p/NRP1 axis. These findings identify circGSE1 as a potential novel therapeutic target for ageing-related cardiovascular diseases.

## Supplementary Material

Supplementary Tables
