# Supplementary material for: Circular RNA circGSE1 promotes angiogenesis in ageing mice by targeting the miR-323-5p/NRP1 axis
Source: Aging (Albany NY). 2022 Apr 1;14(7):3049–69. doi: 10.18632/aging.203988 (PMC9037273; doi:10.18632/aging.203988)
Supplement: Supplementary Tables [file aging-14-203988-s001.pdf]

SUPPLEMENTARY TABLES

Supplementary Table 1. Primers used in this study.

Name	Primer	Sequence(5'-3')
CircGSE1	Forward primer	CGCAAGCTCGCCAAACAG
	Reverse primer	GCCGTGGAAAGCATCCCTAT
Linear GSE1	Forward primer	ATGCTTTCCACGGCGACC
	Reverse primer	TGGGCTCCTCTGCCTGTTT
GAPDH	Forward primer	CAATGACCCCTTCATTGACC
	Reverse primer	TTGATTTTGGAGGGATCTCG
NRP1	Forward primer	GACAAATGTGGCGGGACCATA
	Reverse primer	TGGATTAGCCATTCACACTTCTC
miR323-5p	Stem-loop primer	GTCGTATCCAGTGCAGGGTCCGAGGTATTCGCACTGGATACGACGCGAAC
	Forward primer	AGGTGGTCCGTGGCGC
	Reverse primer	AGTGCAGGGTCCGAGGTATT
miR320-3P	Stem-loop primer	GTCGTATCCAGTGCAGGGTCCGAGGTATTCGCACTGGATACGACTCGCCC
	Forward primer	GCGAAAAGCTGGGTTGAGA
	Reverse primer	AGTGCAGGGTCCGAGGTATT
miR6769b-5P	Stem-loop primer	GTCGTATCCAGTGCAGGGTCCGAGGTATTCGCACTGGATACGACGCTCTT
	Forward primer	CGCCTGGTGGGTGGGG
	Reverse primer	AGTGCAGGGTCCGAGGTATT
miR-6917-5P	Stem-loop primer	GTCGTATCCAGTGCAGGGTCCGAGGTATTCGCACTGGATACGACCGGCAC
	Forward primer	CGCGTGTGGAAGGGGAGTT
	Reverse primer	AGTGCAGGGTCCGAGGTATT
miR-6924-5P	Stem-loop primer	GTCGTATCCAGTGCAGGGTCCGAGGTATTCGCACTGGATACGACACTTCG
	Forward primer	CGAGAGGATGGGGATTTGG
	Reverse primer	AGTGCAGGGTCCGAGGTATT
miR-6943-5P	Stem-loop primer	GTCGTATCCAGTGCAGGGTCCGAGGTATTCGCACTGGATACGACTAGCTT
	Forward primer	GCGTGGGGTGAGGTTGG
	Reverse primer	AGTGCAGGGTCCGAGGTATT
miR-6973a-5P	Stem-loop primer	GTCGTATCCAGTGCAGGGTCCGAGGTATTCGCACTGGATACGACCAACTC
	Forward primer	CGTACGGTGGGAGGGGTG
	Reverse primer	AGTGCAGGGTCCGAGGTATT
miR-7036b-3p	Stem-loop primer	GTCGTATCCAGTGCAGGGTCCGAGGTATTCGCACTGGATACGACGGACCC
	Forward primer	GTTCCTGGTGGGCCTCG
	Reverse primer	AGTGCAGGGTCCGAGGTATT
U6	Stem-loop primer	CTCAACTGGTGTCGTGGAGTCGGCAATTCAGTTGAGAAAAATAT
	Forward primer	CAAGGATGACACGCAAA
	Reverse primer	TCAACTGGTGTCGTGG

Supplementary Table 2. Oligonucleotides and probes used in this study.

Definition	Sequence(5'-3')
si-circGSE1-1	Sense: AGCCCAGAGGCAUGAGCCA<br>Antisense: UGGCUCAUGCCUCUGGGCU
si-circGSE1-2	Sense: CCAGAGGCAUGAGCCAUGA<br>Antisense: UCAUGGCUCAUGCCUCUGG
si-circGSE1-3	Sense: CAGAGGCAUGAGCCAUGAG<br>Antisense: CUCAUGGCUCAUGCCUCUG
si-NRP1-1	Sense: GGGUGCCAUUUCCAAGGAA<br>Antisense: CCCUCGGUAAAGGUUCCUU
si-NRP1-2	Sense: GCUGCAAGAUAACAGAUUA<br>Antisense: CGACGUUCUAUUGUCUAAT
si-NRP1-3	Sense: CCAAGUGAGAAGUGUGAAU<br>Antisense: GGUUCACUCUUCACACUUA
miR323-5p mimic	Sense: AGGUGGUCCGUGGCGCGUUCGC<br>Antisense: GCGAACGCGCCACGGACCACCU
miR323-5p inhibitor	GCGAACGCGCCACGGACCACCU
Cy3-labeled circGSE1 probe	CTCATGCCTCTGGGCTCCTCT
FITC-labeled miR-323-5p probe	CGAACGCGCCACGGACCACCT

Supplementary Table 3. Primary antibodies for western blot and Immunohistochemistry.

Antibody	Company/catalog number
NRP1 (western blot)	Cell Signaling Technology, #3725
NRP1 (Immunohistochemistry)	Abcam,ab81321
CD31(Immunohistochemistry)	Abcam,ab28364
